# The Case for, and Challenges of, Human Cardiac Tissue in Advancing Phosphoprotein Research

**DOI:** 10.3389/fphys.2022.853511

**Published:** 2022-03-23

**Authors:** Amanda W. Huang, Paul M. L. Janssen

**Affiliations:** Department of Physiology and Cell Biology, College of Medicine, The Ohio State University, Columbus, OH, United States

**Keywords:** phosphorylation, human, cardiac, proteins, tissue

## Abstract

Cardiovascular disease (CVD) and stroke affect over 92 million Americans and account for nearly 1 out of 3 deaths in the US. The use of animal models in cardiovascular research has led to considerable advances in treatment and in our understanding of the pathophysiology of many CVDs. Still, animals may not fully recapitulate human disease states; species differences have long been postulated to be one of the main reasons for a failure of translation between animals and humans in drug discovery and development. Indeed, it has become increasingly clear over the past few decades that to answer certain biomedical questions, like the physiological mechanisms that go awry in many human CVDs, animal tissues may not always be the best option to use. While human cardiac tissue has long been used for laboratory research, published findings often contradict each other, leading to difficulties in interpretation. Current difficulties in utilizing human cardiac tissue include differences in acquisition time, varying tissue procurement protocols, and the struggle to define a human “control” sample. With the tremendous emphasis on translational research that continues to grow, research studies using human tissues are becoming more common. This mini review will discuss advantages, disadvantages, and considerations of using human cardiac tissue in the study of CVDs, paying specific attention to the study of phosphoproteins.

## Introduction

### Post-translational Modifications Expand the Function of Proteins

The importance of a protein to a given biological function is not simply reflected by its expression level. Although the total amount of a protein is an important contributing factor to its function, often post-translational modifications (PTMs) are just as or sometimes even more important than its absolute concentration. After completion of the Human Genome Project in 2003 and the start of the Human Proteome Project (HPP) in 2010, there are an estimated 20,000 protein-coding genes that can be transcribed into approximately 150,000 transcripts (Adhikari et al., 2020; Tung et al., 2020). Current research suggests that there are over 400 PTMs (Aebersold et al., 2018), and with PTMs, a protein may have drastically different functions depending on the specific modification. Additionally, multiple PTMs can occur on the same protein and can act in a synergistic or antagonistic manner (Aebersold et al., 2018). Indeed, with PTMs, the sheer number of possible proteins, or proteoforms, climbs exponentially.

With the start of the HPP, much of the interest in PTMs in cardiovascular sciences has been further intensified (Fert-Bober et al., 2018; Adhikari et al., 2020; Reinoso et al., 2021). Commonly studied PTMs in cardiac research include phosphorylation (Ubersax and Ferrell, 2007; van der Velden and Stienen, 2019), ubiquitination (Powell, 2006; Willis et al., 2014), acetylation (Li et al., 2020; Yang et al., 2020), hydroxylation (Markolovic et al., 2015), glycosylation (Laczy et al., 2009; Ngoh et al., 2010; Ng et al., 2021), and nitrosylation (Sun and Murphy, 2010; Murphy et al., 2014). This mini review will focus on phosphorylation in the context of signal transduction in cardiac physiology with special emphasis on research questions that may best be answered using human cardiac tissue.

### Phosphorylation in the Fight or Flight Response

During a potentially life-threatening event, for example encountering a large predator, we have evolved to have a very rapid response that increases our heart rate and force of contraction, allowing us to escape from such a threat (de Lucia et al., 2018). These experiences demand a rapid response from the body, utilizing increased energy consumption and numerous PTMs (de Lucia et al., 2018). Governed largely by β-adrenergic signaling, rapid phosphorylation plays a key role in myocardial signal transduction during the fight-or-flight response (de Lucia et al., 2018). The addition of a phosphate group in the presence of a kinase is a very rapid process on the order of seconds, allowing for swift propagation of signal once there is a stimulus (Gelens and Saurin, 2018). Similarly, the removal of the phosphate group in the presence of a phosphatase is also a rapid process, allowing for rapid signal cessation to conserve energy (Gelens and Saurin, 2018). This process is also enhanced by how kinases, phosphatases, and their targets are located within close proximity to each other (Ubersax and Ferrell, 2007; Mika et al., 2012; Perino et al., 2012).

In addition to how quickly a signal can be initiated and stopped, there are often a greater number of phosphorylatable targets in each succeeding step of the signal transduction cascade, leading to an overall compounding effect (Ubersax and Ferrell, 2007). In other words, cell-wide phosphorylation can occur quickly, spread throughout the cell, and lead to a cell-wide response in a matter of mere seconds. In addition, many proteins have multiple phosphorylation sites, and phosphorylation at each site may have a different functional effect on the protein allows for a very specific fine-tuning response (Ubersax and Ferrell, 2007). Lastly, emerging evidence suggests that phosphorylation, while accepted to sometimes act as an “on-off” switch, can also function as more of a rheostat, in which a particular region of phosphorylatable sites, or a specific level of phosphorylation, results in a graded modification of protein function (Pufall et al., 2005; Landry et al., 2014). In the “rheostat” mode, fine-tuning the function of the protein is achieved through increases and decreases in the number of phosphorylated sites and not necessarily dependent on the specific individual sites themselves (Pufall et al., 2005; Landry et al., 2014). Truly, the fast time frame, the presence of kinases and phosphatases in close vicinity to their targets, and the various modes of control makes phosphorylation an effective choice to yield a precise effect post-stimulus, particularly within the context of cardiac physiology.

### Phosphorylation Is Crucial in the Regulation and Modification of Force and Kinetics

In humans, maintenance of cardiac output is due primarily to frequency-dependent activation and β-adrenergic stimulation, and phosphorylation is critically important in these processes. Mechanisms detailing Frank Starling and force frequency regulation are reviewed here (Frank, 1895; Patterson et al., 1914; de Tombe et al., 2010; Kobirumaki-Shimozawa et al., 2014) and here (Bowditch, 1871; Endoh, 2004).

The overall functional effects of β-adrenergic stimulation in ventricular myocytes are (1) a positive inotropic response (stronger force of contraction leading to greater volume of blood ejected per beat) and (2) an increase in kinetics of contraction and relaxation (shorter time to next contraction or lusitropy allows more beats per unit time). In ventricular myocytes, this is accomplished mainly using the classical pathway involving β-1 receptors; epinephrine that is released binds to the β-adrenergic receptor in the heart, activating the Gs G-protein coupled receptor, leading to activation of adenylyl cyclase (AC). AC produces cAMP from ATP, and one of the main targets of cAMP is protein kinase A (PKA). cAMP binds to the regulatory units of PKA and the catalytic subunits of PKA dissociate and are now active. These catalytic subunits then phosphorylate a large set of proteins that together lead to the functional impacts on contraction and kinetics. The main proteins involved in the cardiac excitation-contraction coupling impacted by PKA activity are listed below, along with the impact phosphorylation has on the function of the protein:

(1) *L-type calcium channel (LTCC):* leads to an increase in the magnitude of the Ca^2+^ current into the sarcolemma (Osterrieder et al., 1982; Reuter, 1983).
(2) *Ryanodine Receptor (RyR)*: leads to increased sensitivity to Ca^2+^-induced activation, which has the functional consequence of increasing the magnitude of Ca^2+^ current released from the sarcoplasmic reticulum (SR) (Hain et al., 1995; Marx et al., 2000; Wehrens et al., 2003).
(3) *Phospholamban (PLB)*: leads to its dissociation from SERCA, thereby increasing the magnitude of Ca^2+^ reuptake from the cytosol back into the SR (Kranias and Solaro, 1982; Lindemann et al., 1983).
(4) *Troponin I (TnI):* leads to decreased Ca^2+^ sensitivity of the thin filament, thereby promoting dissociation of Ca^2+^ and hastening relaxation (Solaro et al., 1976; Moir et al., 1980).
(5) *Myosin Light Chain-2 (MLC-2)*: leads to increased Ca^2+^ sensitivity for force development (Frearson et al., 1976; Holroyde et al., 1979). While it has been shown in transgenic animal studies that MLC-2 is 40% basally phosphorylated, MLC-2 phosphorylation during β-adrenergic response is modest in comparison to other proteins suggesting that the phosphorylation is not a large contributor to positive inotropy in β-adrenergic response.
(6) *Myosin Binding Protein C (MyBP-C)*: abolishes the binding to myosin and weakens the binding to F-actin, thereby accelerating cross-bridge kinetics and increasing the interactions of myosin with the thin filament (Jeacocke and England, 1980; Hartzell and Titus, 1982; Hartzell and Glass, 1984; Garvey et al., 1988).

Combined, these major targets of PKA allow the heart to contract stronger and faster, drastically increasing cardiac output.

### The Critical Need for Studies Involving Human Cardiac Tissue

Ultimately, most research, especially that funded by health organizations, is to improve the health and understanding of human diseases. The goal is not necessarily to discover mechanisms underlying heart failure progression in a transgenic, genetically, and environmentally homogeneous animal but rather the impact in a human population. Indeed, stark differences that greatly impact contraction and relaxation between human and animal models have been reported (Milani-Nejad and Janssen, 2014), and animal models to study certain aspects of CVDs, namely physiology and the subsequent physiological changes that result due to different diseases processes, do indeed fall short of describing the human condition (Milani-Nejad and Janssen, 2014). In the case for the status of phosphoproteins in cardiac tissue, while myofilament calcium sensitivity is increased in both mouse models of and human heart failure, the magnitude of the shift is much less pronounced in rodents than in humans (Marston and de Tombe, 2008). This shift is hypothesized to be due to increased basal levels of phosphorylation in rodents (Hamdani et al., 2008). From this finding, decreasing myofilament calcium sensitivity may have a greater and more beneficial impact on human HF rather than animal HF, a hypothesis that may be worth pursuing when studying mechanisms to treat human HF but may appear less attractive when only considering the animal studies that were conducted. Clearly, functional findings from small rodents should be validated in human tissue. Utilizing human tissue can help bridge the gap between animal studies completed in genetically and environmentally similar animals and human clinical trials consisting of large numbers of humans with genetically unique and environmentally diverse lives.

### Cardiac Tissue Collection Processes Differ From Animals and Humans

The studies conducted to elucidate the role of phosphoproteins in cardiac physiological mechanisms such as β-adrenergic stimulation discussed above were conducted primarily in animals. However, tissue collection processes for animal and human cardiac tissue often differ in multiple and sometimes critically important aspects with important implications for research questions involving phosphoproteins.

### Surgery

Since the 1930s, surgery has been recognized as a major physiologic stressor, involving a complicated interplay among 3 major systems: the sympathetic, endocrine, and immune systems, ultimately culminating in the surgical stress response (Cuthbertson, 1932; Desborough, 2000; Cusack and Buggy, 2020). The start of surgery results in an abrupt rise in serum cortisol levels, various hormones secreted from the pituitary, insulin resistance, cytokine production, the initiation of the acute phase reaction, as well as proliferation of neutrophils and leukocytes (Desborough, 2000; Cusack and Buggy, 2020). The combined effect results a state where the body breaks down body fuel and retains water (Desborough, 2000; Cusack and Buggy, 2020). In addition, the invasiveness of the surgery (i.e., size and depth of incisions) and the length of time of the surgery have all been documented to influences these systems proportionally (Desborough, 2000; Cusack and Buggy, 2020). Indeed, it has been found that laparoscopic surgeries, since the severity of tissue wounding is less than conventional procedures, result in less of an increase in concentrations of pro-inflammatory immune mediators like the acute phase protein C-reactive protein (CRP) and the cytokine IL-6 (Kehlet, 1997). In the clinic, minimally invasive procedures often allow for faster discharge and recovery times. Selection of the appropriate anesthetics to counter the length and intensity of the surgical stress response is a foundational block of anesthesiology training (Cusack and Buggy, 2020).

Because of this surgical stress response, sham surgeries are performed in animals on the control group to control for the confounding effect of surgery itself on the experimental design. For example, to study heart failure in a pressure overload murine model, mice in the diseased group undergo aortic banding surgeries while those in the non-diseased group undergo sham surgeries. However, the actual surgical procedures to obtain diseased and non-diseased human cardiac tissue are vastly different. Researchers, when studying hypertrophic cardiomyopathy (HCM), can obtain samples during a surgical septal myectomy (Septal Myectomy, 2019). These procedures involve complete sedation of the patient using a cocktail of 3 or 4 inhaled and intravenous anesthetics, complete exposure of the thoracic cavity by cutting through the sternum, and placement of the patient on cardiopulmonary bypass, ultimately resulting in a total procedure time of 3–6 h (Septal Myectomy, 2019). To obtain non-diseased control cardiac samples, researchers commonly obtain samples during cardiac catherization. Cardiac catherizations are endovascular procedures that do not require cardiopulmonary bypass and only requires mild to moderate sedation unlike deep sedation that is necessary for surgical myectomies (Cardiac Catherization, 2019). Most patients are sedated but awake and conscious during the entire procedure (Cardiac Catherization, 2019). In addition, cardiac catherizations, with an experienced interventional cardiologist, can be completed in about 30 min (Cardiac Catherization, 2019). Tissue acquisition during a cardiac catherization is very appealing due to the high number of procedures that occur in a day. Therefore, there is a high possibility in receiving enough samples to achieve a researcher’s desired group size. However, the cardiac catherization is truly different from the ventricular myectomy to be considered as a control procedure for this case. A lengthier, open-heart surgery that involves general anesthesia, for example an organ procurement, would be a better control procedure for a ventricular myectomy. Future studies directly comparing PTMs in non-diseased samples between cardiac catherization and whole organ procurement may shed light on the effect size of these procedures.

Factors such as depth and length of anesthesia, additional medications administered, and length of surgery time cannot be controlled by researchers in their study design, as these are typically dictated by patient and case-specific needs. However, selecting samples from surgeries with similar components (open heart vs. not, general anesthesia vs. not, similar lengths of time) is under a researcher’s control. Importantly, an analysis into what kind of procedure will be utilized to obtain diseased and control tissue prior to the start of the study can minimize the probability that a subsequent effect (or lack thereof) seen with these tissue studies is due to the surgery itself.

### Time

It is standard practice for animal studies to be conducted immediately post-sacrifice to minimize cell death and tissue loss. As one of the studies that identified TnI to be phosphorylated with beta-adrenergic stimulation, Solaro et al. (1976) conducted their experiments by stunning rabbits, rapidly excising their intact hearts, cooling with saline, and perfusing with a buffer in a recirculation chamber. More recently, in the mouse heart, phosphorylated GSK and CREB were found to be significantly decreased at 10 and 5 min post-mortem (Wang et al., 2015). In addition, the extent of dephosphorylation has been shown to depend on site, protein, and tissue studied (Li et al., 2003; Wang et al., 2015).

While in many studies human cardiac tissue is not obtained post-death, a concept similar to the post-mortem interval called “cold ischemic time” applies. Cold ischemic time is defined as the time elapsed between aortic cross clamping of the donor heart and reperfusion of said heart in the recipient. It has been shown that in humans, extended cold ischemic time (defined as greater than 6.25 h) in those receiving a heart transplant aged 34 and older was associated with decreased median survival of more than 1,000 days (2.74 years) (Russo et al., 2007). From this, it follows that increased cold ischemic time for research, or time between aortic cross clamping and the start of physiologic experiments, may also impact the viability of tissue for physiological research questions that involve active contraction and relaxation.

Unfortunately, in human tissue collection, time delays between excision of sample and acquisition by the research team are inevitable. There are many common and probable time delays. Firstly, if research team members are not allowed to be in the sterile field of the operation, which they often are not, they will not be able to retrieve the sample directly from the surgeon. In this case, researchers would instead have to wait for additional surgical team members to give them the specimen. A closely associated case is that the specimens may be handled by those who are not familiar with or trained on research protocols, protocols that may differ drastically from routine clinical practice. For example, while storage of cardiac biopsies in saline or minimal volumes of solution may be acceptable for certain pathological tests, this method of handling would not be acceptable for many physiological experiments, as exposure to air in the absence of solution drastically reduces the viability of the tissue. Lastly, there will inevitably be some varying amount of time to travel from the operating room (OR) back to the laboratory to begin experiments, with minimal amount of travel time from a hospital to a laboratory on the same campus and varying amount of travel time from a hospital to a laboratory being located at distances that would require a car or even air transportation. All the aforementioned instances increase the cold ischemic time.

Historically, since there has not been a defined protocol for tissue procurement, human cardiac tissue collection has resulted in variable tissue procurement strategies, if even mentioned at all in the methods sections of published papers. Such variable tissue procurement strategies and often conflicting conclusions derived from them led to multiple investigations into the differing effect of tissue procurement strategies on the phosphorylation status of myofibrillar proteins (Noguchi et al., 2004; Jweied et al., 2007; Walker et al., 2011). Of the procurement strategies that have been described, two commonly utilized strategies involve flash freezing in the OR and cardioplegic perfusion after excision. While flash freezing in the OR minimizes cold ischemic time and preserves the tissue in a phosphorylation state very similar to that right before excision, it precludes the functional study of live tissue in physiologic apparatuses. However, physiologic measurements from skinned muscle fibers are still a viable option from these tissues. Additionally, storage at −80C does not preserve phosphorylation status indefinitely, as storage of myofilament preparations for longer than 30 days at −80C was found to lead to a global decrease in phosphorylation levels (Utter et al., 2015). While use of additional cardioplegia may allow researchers some flexibility in time, there have been reports that suggest that prolonged exposure in cardioplegia may lead to dephosphorylation, although length of time in cardioplegia and whether there was adequate washout of the cardioplegia were not discussed (Jweied et al., 2007). It remains unanswered if the dephosphorylation seen was result of tissue procurement protocols or from the natural recovery from the surgical stress response. Ultimately, many cardiac procedures, for example heart transplantation, involve a cardioplegic flush post cross-clamp, so if researchers would like to acquire human cardiac tissue during surgery, the use of cardioplegia and its effects are inevitable (Liao and Shumway, 2014).

With the interplay of these complicated factors, procurement strategies should be tailored to the research question of interest (Gupta et al., 2012). One method of procurement may not be acceptable for a different question of interest. In research questions involving phosphoproteins, cardiac tissue collected will most likely reflect the status of phosphoproteins right before a method of preservation, such as flash freezing in the OR to preserve phosphoprotein status right after excision. Therefore, considerations for the surgery and physiological conditions prior to the excision are very important. It would be important to select surgical procedures in similar lengths of time as the longer the surgery, the greater the stressor, and the potential for a more profound effect on the beta-adrenergic system (Desborough, 2000; Cusack and Buggy, 2020). Additionally, different anesthetics have been suggested to affect the lability of the phosphate group of certain cardiac myofilament proteins, but the effect is anesthetic and myofibrillar protein-specific (Solaro et al., 1976; Utter et al., 2015). If one wishes to ultimately study contractile cardiac tissue, tissue procurement strategies similar to those for preserving donor hearts for subsequent transplantation are suggested, including maintaining the heart in a hypothermic environment, use of cardioplegia administered through major coronary vessels to arrest the heart, and storage in preservation solutions within a cooler with ice (Chambers and Fallouh, 2010; Liao and Shumway, 2014). Lastly, improved preservation of donor heart function is a continuing area of active research in cardiac surgery (Ardehali et al., 2015).

### Limitations: Time

Several groups have made great strides in procuring human cardiac tissue as quickly as possible (dos Remedios et al., 2017; Chung et al., 2018b; Mashali et al., 2021). Even though procurement in the OR occurs within minutes, phosphorylation takes place within mere seconds. Hence, future directions in which human cardiac tissue can be procured at a certain physiologic state within seconds is still an ongoing battle. Given the logistics and the need to minimize patient risks, further reducing the time between excision and procurement will be paramount for studies, specifically for phosphorylation, where procurement within minutes may not be sufficient. Although many projects would be greatly enhanced by tissue collected within minutes, studies in phosphorylation utilizing human cardiac tissue may warrant a different approach.

While there are certain periods of time that cannot be controlled by researchers (i.e., length of time to excise the sample, walking and driving times between OR and laboratories), selecting methods and establishing protocols to ensure that the tissue collected is in a viable state for the intended experiment can increase the chance of success of experiments.

### A Tissue Procurement Strategy: The Ohio State University Human Heart Resource

There are many centers across the US and the world that procure human cardiac tissue for basic, clinical, and translational research, and these groups have published protocols for the establishment of a biobank and procurement of human cardiac tissue (Yamada et al., 2013; Lal et al., 2015; Blair et al., 2016). Many of these centers have similar components: acquisition of tissue and clinical information. Currently, we are one of the few centers in the United States that is able to procure and study live, contracting tissue from failing and non-failing hearts. Through our partnership with the Division of Cardiac Surgery at The Ohio State University Wexner Medical Center, we have procured approximately 10–15 end-stage failing human hearts per year, while from Lifeline of Ohio (LOOP), approximately 10–20 hearts per year. Over the past decade, we have procured over 200 hearts and have established procurement protocols that allows for nearly identical procurement and transport of failing and non-failing heart (Chung et al., 2018b; Mashali et al., 2021). Being present in the operating room during the time of the procedures allows for rapid processing within minutes. Seconds after the heart is excised from the body, it is perfused with 4°C cardioplegic solution before being submerged in cardioplegia to be transported in a cooler packed with ice. Once back to the laboratory, samples are immediately flash frozen in liquid nitrogen. In addition to access to freshly procured tissue, we have access to the clinical history and biometrics for all our procured hearts, allowing to control, to the best of our ability, for age, gender, race, comorbidities, as well as acute and long-term pharmacotherapies.

### Concerns With “Control” Human Cardiac Tissue Samples

The question of how closely control donor samples differ from each other as well as how closely they represent a truly non-diseased individual has been raised (Jweied et al., 2007). From the onset of our tissue procurement program in 2010, we have published multiple studies on contractile function utilizing human cardiac tissue. Baseline values of twitch force and kinetics values are highly similar among all our studies (Milani-Nejad et al., 2016, 2018; Chung et al., 2018a,b; Janssen et al., 2018; Mashali et al., 2021). These findings suggest that having a standard protocol with similar procurement times does result in reliable physiological data with close similarities even with low *n* numbers.

Other groups, such as Marston et al. (2020), also studied this criticism and found similar results. Utilizing control human cardiac tissue samples collected from 1994 to 2011 from the Sydney Heart Bank, Marston et al. (2020) conducted *in vitro* motility and TnI phosphorylation measurements. All donors were brain dead and were organ donors, and while time on life support and cardioplegia varied as well, the *in vitro* motility measurements and TnI phosphorylation measurements of the samples were similar to each other, no matter what the specific cause of death was. The group also obtained other donor cardiac samples from other groups that used similar acquisition techniques as those from the Sydney Heart Bank and found similar measurements across all samples. While it can be argued that perhaps the results would be different had Marston’s research team conducted other assays, it is undoubtedly a strength that for 2 commonly used assays utilized in human cardiac tissue research, the measurements in the samples were rather comparable.

### Alternative Strategies

When it is not possible to procure tissue right in the operating room, an alternative strategy would the establishment of a physiologic homeostasis *ex vivo.* Once established, this could be followed by subsequent flash freezing of the preparation for phosphoprotein analyses. Several experimental systems have been developed that can accomplish this, systems in which tissue can be kept contracting for days up to months (Janssen et al., 1999; Fischer et al., 2019). Mammalian, including human, trabecular myocardial preparations were kept isometrically contracting under physiologic stimulation, pH, and temperature for up to 6 days (Janssen et al., 1999). Such preparations exhibited no significant decrease in active developed force and diastolic force, and the force frequency and β-adrenergic responses were intact and preserved (Janssen et al., 1999). These muscle preparations can also be subjected to non-isometric conditions, for investigation of remodeling and mechanics (Guterl et al., 2007), as well as gene transfer and subsequent analysis (Lehnart et al., 2000; Janssen et al., 2002). More recently, Fischer et al. (2019) showed that precision-cut human myocardial slices from explanted failing human hearts could be maintained in biomimetic tissue culture dishes. In these biomimetic chambers, slices were maintained under a physiologic preload as well as continuously electrical stimulated whilst preserved for up to 4 months or 2,000,000 beats *in vitro* (Fischer et al., 2019). In addition to being a tool for studying long term drug effects on human myocardium, these myocardial slices as well as the trabecular culture can also be flash frozen at different physiological conditions and subsequently used for phosphoprotein analyses. Lastly, if human cardiac tissue is unable to be procured, the burgeoning field of human engineered cardiac tissues (hECTs) offer additional potential solutions (Weinberger et al., 2017; Campostrini et al., 2021). Of particular interest, hECTs that are scaffold based engineered tissues can contract against loads and be manipulated under different physiologic conditions (Campostrini et al., 2021). More recently, engineered heart tissues were created to form a novel *in vitro* model system, and through subsequent slacking and stretching processes, was able to provide new mechanistic insights into reverse remodeling post-mechanical unloading (Shen et al., 2021). Truly, the flourishing field of hECTs has led to the development of *in vitro* models that mimic physiological states and therefore show great promise to be used to study phosphoproteins underlying different physiological states.

### Research Is a Secondary Goal in Studies Involving Humans

In research studies, animals are bred and utilized primarily for the role of carrying out a specific experimental design. However, researchers utilizing human cardiac tissue must understand that the patient’s health and treatment of his or her diseases is paramount. If there is a need for such treatments during the surgery or any other interventions, they will be utilized because the patient’s wellbeing and hemodynamic stability cannot be compromised. Because of this, there are so many potentially confounding variables that researchers cannot control for.

Researchers will not be able to have the same level of detailed control with human tissues like they have in animal studies. Historically, this has been dealt with in human studies using large sample sizes to combat the effect of these confounds and variability. Indeed, the number of humans typically enrolled for a given clinical trial is in the thousands, but in basic animal laboratory research, only a few dozen mice are needed for animal studies. However, each variable we can control for, or minimize impact of, in human tissue research therefore has the potential of greatly reducing the sample size to more manageable numbers: research on several tens of patient samples, rather than thousands, making the utilization of human cardiac tissue samples and the ability to draw strong conclusions from them more of a norm and less of an exception.

## Conclusion

While animal models have led to tremendous gains in knowledge of the pathophysiology of CVDs and the development of many current therapies that are improving the lives of millions, it has also become clear that depending on certain research questions, the sole use of animal models and the results from those studies alone are incomplete. The use of human cardiac tissue for research is hampered by differences in tissue acquisition times and often the lack of a control group but these concerns should not deter investigators from utilizing such tissue. Certain methods of tissue procurement are acceptable for certain downstream applications and should be considered by researchers when developing a question. Additionally, there are at least a few cardiac tissue banks that procure healthy donor hearts for comparison as a control group in studies. By increasing the use of human tissue in cardiovascular research and taking that along with the breadth of knowledge we have gained from animal models, we will be better able to draw translatable conclusions to better understand disease processes and to design therapies to improve human health.

## Author Contributions

PJ conceptualized the review. AH conducted the literature search and wrote the initial draft. Both authors critically revised and approved the final manuscript.

## Conflict of Interest

The authors declare that the research was conducted in the absence of any commercial or financial relationships that could be construed as a potential conflict of interest.

## Publisher’s Note

All claims expressed in this article are solely those of the authors and do not necessarily represent those of their affiliated organizations, or those of the publisher, the editors and the reviewers. Any product that may be evaluated in this article, or claim that may be made by its manufacturer, is not guaranteed or endorsed by the publisher.
